# Supplementary material for: An Interplay between Viruses and Bacteria Associated with the White Sea Sponges Revealed by Metagenomics
Source: Life (Basel). 2021 Dec 24;12(1):25. doi: 10.3390/life12010025 (PMC8777954; doi:10.3390/life12010025)
Supplement: Supplementary file 1 [file life-12-00025-s001.zip › life-1483063-supplementary.pdf]

life

MDPI

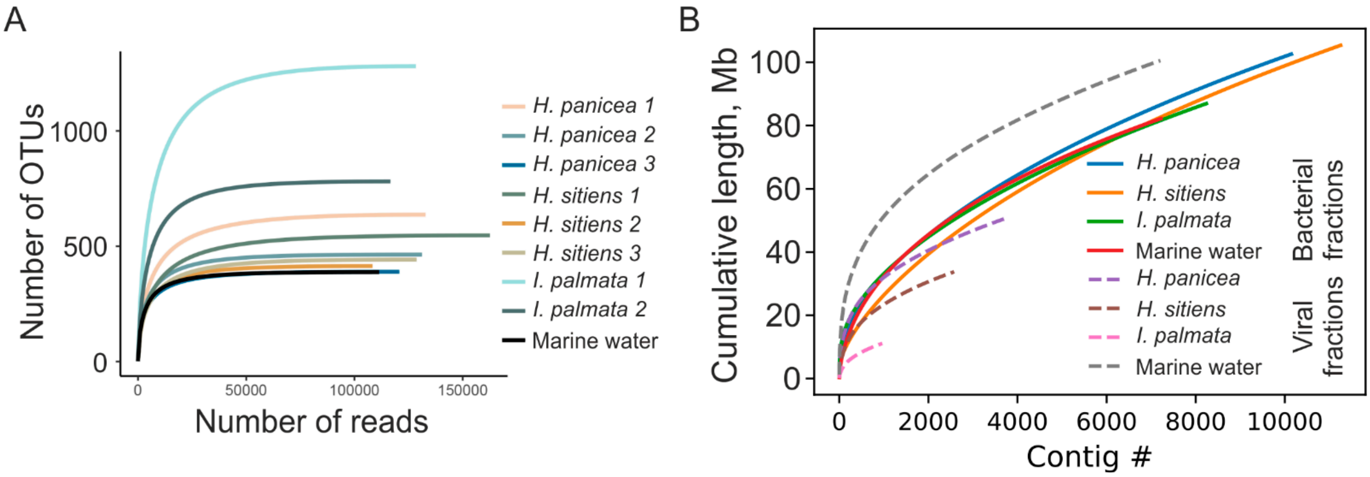

Figure S1. (A) Rarefaction curves analysis of V3-V4 16S metagenomes sequences in this study. (B) Analysis of the cumulative length of metagenomes for contigs > 5 kb.

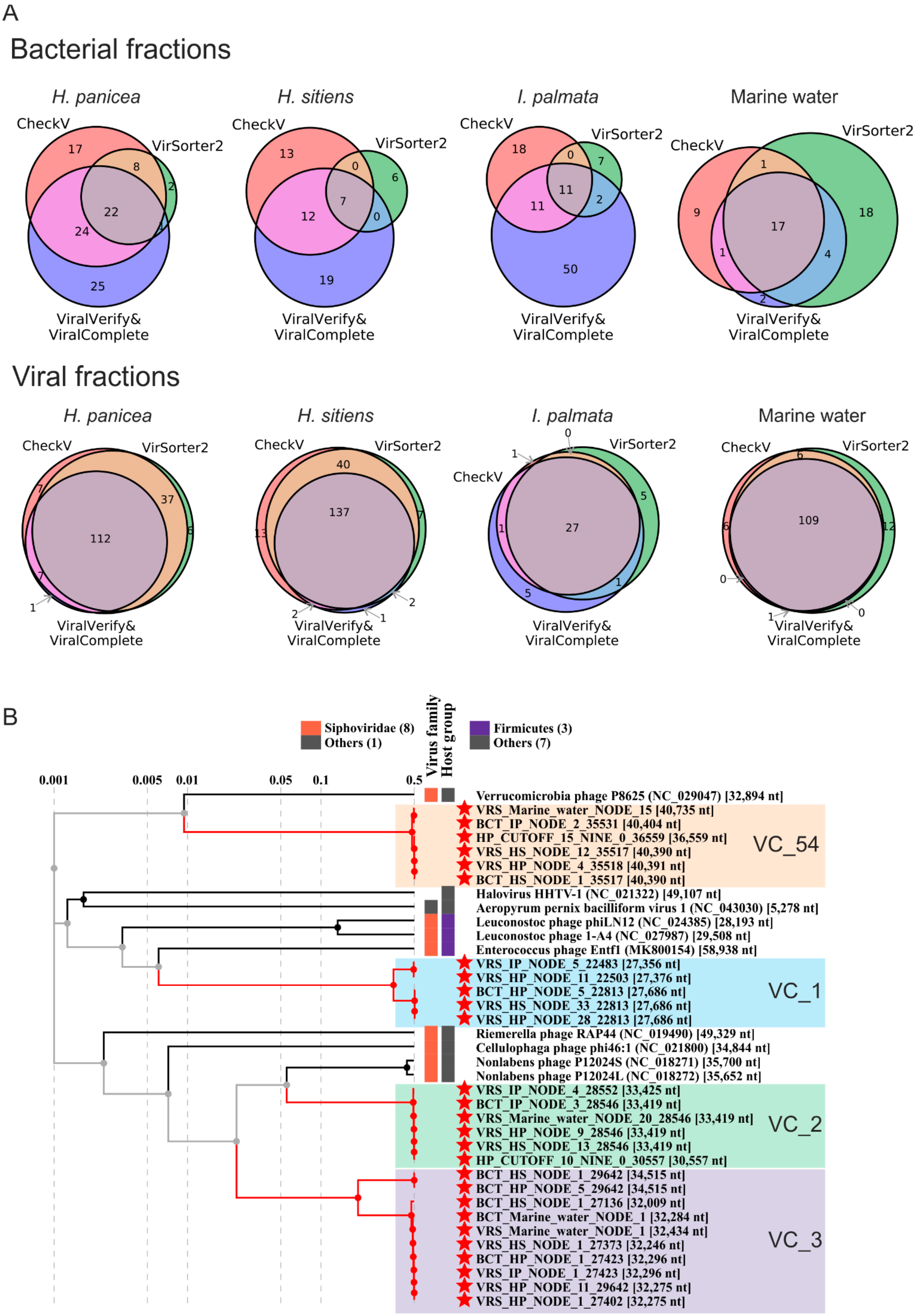
A
Bacterial fractions
H. panicea
CheckV
VirSorter2
ViralVerify&
ViralComplete
17
8
2
22
24
1
25
H. sitiens
13
0
6
7
12
0
19
I. palmata
18
0
7
11
11
2
50
Marine water
1
9
17
18
1
4
2
Viral fractions
H. panicea
7
37
112
6
7
1
H. sitiens
40
137
7
13
2
1
2
I. palmata
0
1
5
27
1
1
5
Marine water
6
109
6
12
0
1
0
B
Siphoviridae (8)
Others (1)
Virus family
Host group
Firmicutes (3)
Others (7)
0.001
0.005
0.01
0.05
0.1
0.5
Verrucomicrobia phage P8625 (NC_029047) [32,894 nt]
VRS_Marine_water_NODE_15 [40,735 nt]
BCT_IP_NODE_2_35531 [40,404 nt]
HP_CUTOFF_15_NINE_0_36559 [36,559 nt]
VRS_HS_NODE_12_35517 [40,390 nt]
VRS_HP_NODE_4_35518 [40,391 nt]
BCT_HS_NODE_1_35517 [40,390 nt]
VC_54
Halovirus HHTV-1 (NC_021322) [49,107 nt]
Aeropyrum pernix bacilliform virus 1 (NC_043030) [5,278 nt]
Leuconostoc phage phiLN12 (NC_024385) [28,193 nt]
Leuconostoc phage 1-A4 (NC_027987) [29,508 nt]
Enterococcus phage Entf1 (MK800154) [58,938 nt]
VRS_IP_NODE_5_22483 [27,356 nt]
VRS_HP_NODE_11_22503 [27,376 nt]
BCT_HP_NODE_5_22813 [27,686 nt]
VRS_HS_NODE_33_22813 [27,686 nt]
VRS_HP_NODE_28_22813 [27,686 nt]
VC_1
Riemerella phage RAP44 (NC_019490) [49,329 nt]
Cellulophaga phage phi46:1 (NC_021800) [34,844 nt]
Nonlabens phage P12024S (NC_018271) [35,700 nt]
Nonlabens phage P12024L (NC_018272) [35,652 nt]
VRS_IP_NODE_4_28552 [33,425 nt]
BCT_IP_NODE_3_28546 [33,419 nt]
VRS_Marine_water_NODE_20_28546 [33,419 nt]
VRS_HP_NODE_9_28546 [33,419 nt]
VRS_HS_NODE_13_28546 [33,419 nt]
HP_CUTOFF_10_NINE_0_30557 [30,557 nt]
VC_2
BCT_HS_NODE_1_29642 [34,515 nt]
BCT_HP_NODE_5_29642 [34,515 nt]
BCT_HS_NODE_1_27136 [32,009 nt]
BCT_Marine_water_NODE_1 [32,284 nt]
VRS_Marine_water_NODE_1 [32,434 nt]
VRS_HS_NODE_1_27373 [32,246 nt]
BCT_HP_NODE_1_27423 [32,296 nt]
VRS_IP_NODE_1_27423 [32,296 nt]
VRS_HP_NODE_11_29642 [32,275 nt]
VRS_HP_NODE_1_27402 [32,275 nt]
VC_3

Figure S2. (A) The Venn diagrams represent the concordance between the predictions of viral sequences by ViralVerify & ViralComplete pipeline, VirSorter2, and CheckV. Numbers are given for sequences before clustering with Cd-hit. Sequences detected by at least two tools were defined as putative viral contigs and selected for further analyses. (B) Phylogenetic tree of representative viral contigs (indicated by red asterisks) belonging to 4 the most abundant viral clusters composed exclusively of viral contigs from the White Sea metagenomes. The tree was prepared using ViPTree. Viral cluster's IDs correspond to the IDs indicated on the network in Figure 3C. Branch length is a genomic similarity score distance.

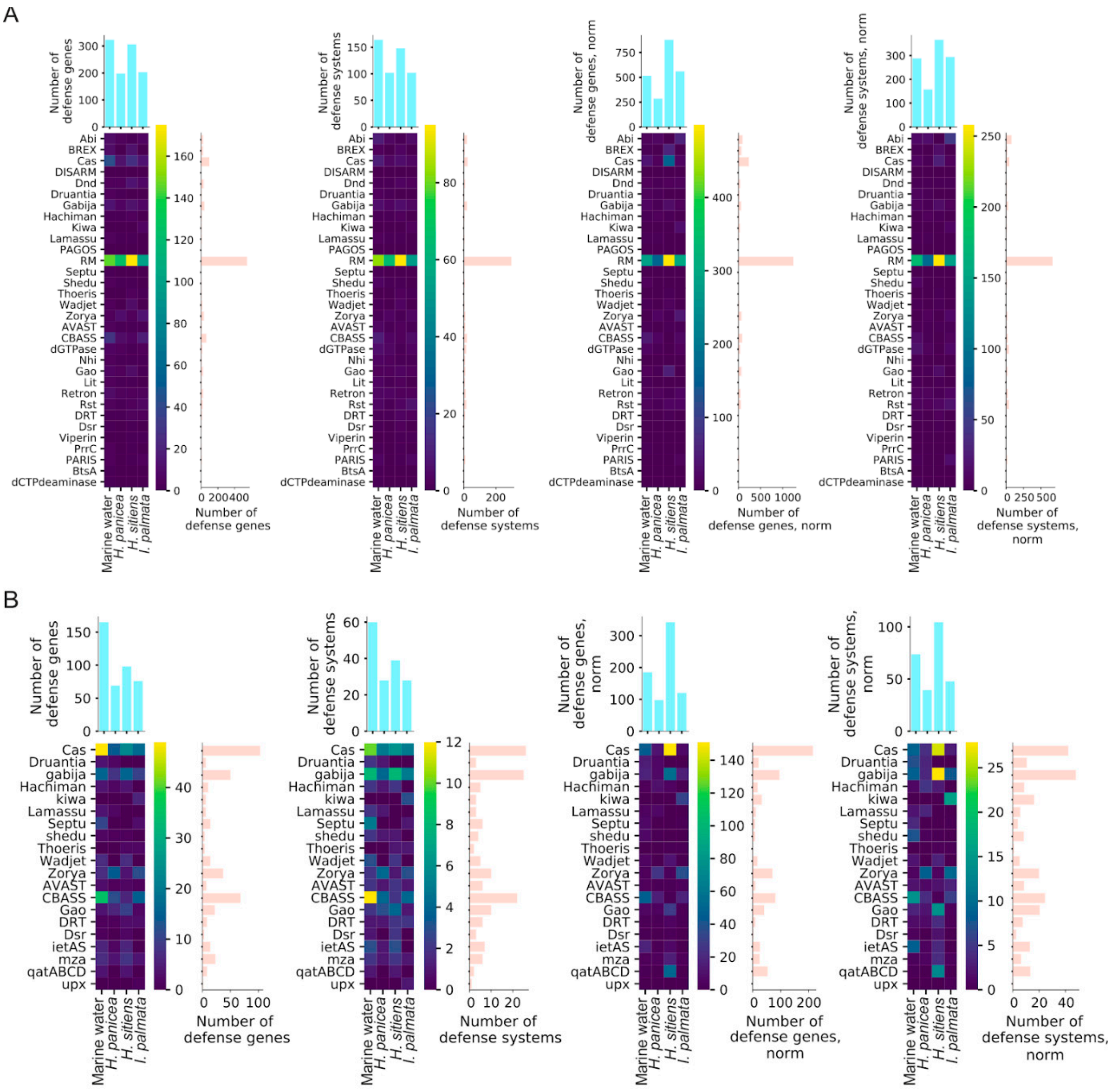

Figure S3. (A) Heatmaps representing, from left to right, the numbers of defense systems genes, numbers of putative defense systems, normalized numbers of defense systems genes, and normalized numbers of putative defense systems found in metagenomes using DefenseFinder. (B) Heatmaps representing, from left to right, the numbers of defense systems genes, numbers of putative defense systems, normalized numbers of defense systems genes, and normalized numbers of putative defense systems found in metagenomes using PADLOC.

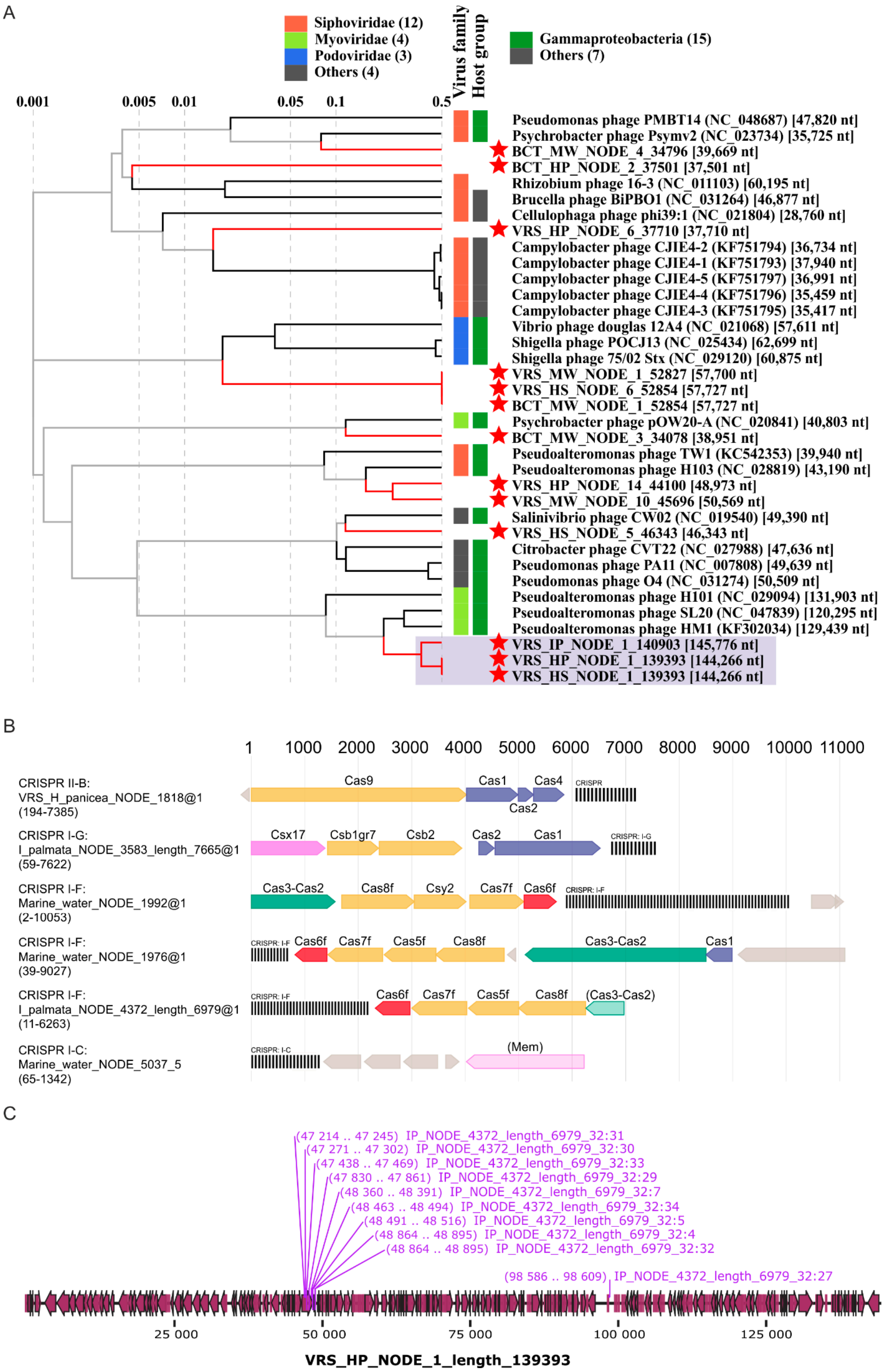

Figure S4. (A) Phylogenetic tree of viral sequences constructed with ViPTree. Putative viral contigs with detected protospacers are highlighted by red asterisks. The blue rectangle marks the group of related contigs found in all sponges, containing protospacers matching by several spacers of the I-F CRISPR-Cas system from the *I. palmata* metagenome. Branch length is a genomic similarity score distance. (B) CRISPR-Cas systems containing spacers for which matching protospacers were found in the putative viral contigs. Arrays are in alternating black/white displaying the actual number of repeats/spacers, and with their predicted subtype association based on the consensus repeat sequence. The interference module is in yellow. Cas6 is in red. Accessory genes are in purple Genes with alignment scores below the thresholds are lighter and with parentheses around names. Unknown genes are in gray. (C) Annotation of a viral contig targeted by 10 spacers (positions of protospacers are labeled) from I-F CRISPR-Cas system identified in the *I. palmata* metagenome.
